# PLCL/SF/NGF nerve conduit loaded with RGD‐TA‐PPY hydrogel promotes regeneration of sciatic nerve defects in rats through PI3K/AKT signalling pathways

**DOI:** 10.1111/jcmm.18544

**Published:** 2024-08-04

**Authors:** Kunyu Liu, Weilong Tang, Shixin Jin, Xin Hao, Yuhang Hu, Tianyi Zhou, Chenliang Zhou, Guanghua Chen, Yifeng Cui, Qianqi Liu, Zhenyu Zhang

**Affiliations:** ^1^ Department of Orthopedic The First Affiliated Hospital of Harbin Medical University Harbin China; ^2^ Department of Orthopedic The Second Affiliated Hospital of Harbin Medical University Harbin China; ^3^ Department of Hepatic Surgery The First Affiliated Hospital of Harbin Medical University Harbin China; ^4^ Department of Ultrasound The First Affiliated Hospital of Harbin Medical University Harbin China

**Keywords:** conductive hydrogel, nerve conduit, PI3K/AKT, PLCL/SF/NGF@TA‐PPy‐RGD, sciatic nerve defects

## Abstract

Peripheral nerve defect are common clinical problem caused by trauma or other diseases, often leading to the loss of sensory and motor function in patients. Autologous nerve transplantation has been the gold standard for repairing peripheral nerve defects, but its clinical application is limited due to insufficient donor tissue. In recent years, the application of tissue engineering methods to synthesize nerve conduits for treating peripheral nerve defect has become a current research focus. This study introduces a novel approach for treating peripheral nerve defects using a tissue‐engineered PLCL/SF/NGF@TA‐PPy‐RGD conduit. The conduit was fabricated by combining electrospun PLCL/SF with an NGF‐loaded conductive TA‐PPy‐RGD gel. The gel, synthesized from RGD‐modified tannic acid (TA) and polypyrrole (PPy), provides growth anchor points for nerve cells. In vitro results showed that this hybrid conduit could enhance PC12 cell proliferation, migration, and reduce apoptosis under oxidative stress. Furthermore, the conduit activated the PI3K/AKT signalling pathway in PC12 cells. In a rat model of sciatic nerve defect, the PLCL/SF/NGF@TA‐PPy‐RGD conduit significantly improved motor function, gastrocnemius muscle function, and myelin sheath axon thickness, comparable to autologous nerve transplantation. It also promoted angiogenesis around the nerve defect. This study suggests that PLCL/SF/NGF@TA‐PPy‐RGD conduits provide a conducive environment for nerve regeneration, offering a new strategy for peripheral nerve defect treatment, this study provided theoretical basis and new strategies for the research and treatment of peripheral nerve defect.

## 1 INTRODUCTION

The peripheral nerves refer to nerves outside the central nervous system (brain and spinal cord), which can receive sensory impulses from other tissues in the body and transmit this information to the brain in the form of electrical pulses along nerve fibres.^1^ The motor commands generated in the brain were sented to the nerves along rapidly transmitting nerve fibres, and the generated electrical signals directly coordinate muscle contraction movements through the peripheral nerves.^2^ Peripheral nerve injury (PNI) is a common clinical problem caused by trauma or other diseases, affecting millions of people worldwide each year and often leading to loss of sensory and motor function in patients.^3^

Peripheral nerve injury is usually caused by traumatic, non traumatic, or iatrogenic events. The development of painful neuropathy, neuroma, sensory disorders, decreased muscle strength, and paralysis in patients is all caused by damage and loss of sensory and motor nerves. Due to the motor and functional impairments caused by peripheral nerve injury, it has not only inflicted great physical and mental trauma on the patients themselves but also brought significant economic and psychological burdens to families and society. Therefore, peripheral nerve injury has become a concern of the whole society.^4^ Additionally, peripheral nerve injury secretes inhibitory factors that prevent the growth of myelin sheaths and neurons, and cannot express necessary growth factors, forming an inhibitory environment and dense scar tissue around the injury site.^5^ How to construct a favourable microenvironment for nerve regeneration and functional recovery after peripheral nerve injury has become a critical and urgent problem in scientific research and clinical treatment. The main mechanism of peripheral nerve injury repair includes promoting functional nerve regeneration and establishing connections.^6^ Currently, research on the treatment of peripheral nerve injury mainly includes nerve growth stimulating factors or promoting nerve protection and regeneration, and tissue engineering scaffolds bridging neuropathic areas to promote endogenous nerve growth.^7^ There are already many effective therapies for PNI in clinical practice. For most PNI caused by trauma, surgical exploration and treatment are required. The clinical methods used to repair peripheral nerve defects mainly include nerve stump anastomosis,^8^ artificial nerve conduit,^9^ and autologous nerve transplantation.^10^ Autologous nerve transplantation is the preferred option, but its clinical application is limited due to insufficient donor tissue, the possibility of forming neuromas and losing nerve function.^11^ In recent years, the application of tissue engineering methods to synthesize neural conduits for the treatment of peripheral nerve defects has become a research hotspot. To overcome the above limitations, neural tissue engineering scaffolds based on biomimetic strategies have become a reliable alternative to nerve transplantation in clinical practice. Traditional tissue engineering focuses on using natural or synthetic materials to prepare nerve guide conduits (NGCs) for the treatment of PNI. NGCs serve as bridges between the severed ends of damaged nerves, supporting axonal regeneration along the conduit.^12^, ^13^

An ideal neural conduit should have the following characteristics: good mechanical properties, high porosity, good biocompatibility, and conductivity. Chitosan, collagen, poly(glycolic acid) (PGA), poly(hydroxybutyrate‐co‐hydroxyvalerate) (PHBV)‐poly(lactic‐co‐glycolic acid) (PLGA), and polyvinyl alcohol (PVA) are biocompatible materials that have been extensively researched and applied in the development of nerve conduits for neural tissue engineering. Chitosan, derived from the exoskeleton of crustaceans, is known for its biocompatibility, biodegradability, and ability to promote cell adhesion and proliferation, making it an excellent material for nerve conduits. Studies have shown that chitosan conduits significantly enhance peripheral nerve regeneration by providing an optimized environment conducive to axonal growth.^14^ Collagen, being the most abundant protein in mammals, stands out for its natural biocompatibility and its role in promoting cellular interactions and tissue development. Its application in nerve conduits is particularly beneficial, owing to its exceptional mechanical properties and its ability to support cell attachment, migration, and differentiation, all of which are essential for nerve repair.^15^ PGA is a synthetic, biodegradable polymer known for its hydrolytic degradation properties, allowing for the adjustment of degradation rates. Conduits made from PGA are valued for their high tensile strength and their support of cell attachment and proliferation, which are critical for sustaining regenerating nerve fibres.^16^ The PHBV–PLGA composites combine the advantageous properties of PHBV and PLGA, offering adjustable mechanical properties and degradation rates. This combination provides a versatile platform for designing nerve conduits that can closely mimic the natural extracellular matrix, thereby supporting various stages of neural tissue regeneration.^17^

PVA is a synthetic polymer recognized for its excellent biocompatibility and hydrophilic properties, making it particularly suitable for creating hydrogel‐based nerve conduits. These hydrogels provide a soft, supportive scaffold that simulates the natural neural tissue environment, thereby promoting nerve growth and functional recovery. The unique characteristics of each material, such as promoting cellular adhesion, proliferation, and differentiation, while providing mechanical support and guidance for regenerating nerve fibres, are crucial for the advancement of nerve regeneration techniques.^18^ However, traditional NGCs have a hard and inflexible structure, often leading to mismatch with neural tissue and the risk of local pruning of nerve stumps.^19^ Many researchers are committed to developing new treatment strategies for PNI. In recent years, based on the inherent physical, chemical and biological characteristics of hydrogels, they have been widely used in biomedical materials such as drug and gene delivery, contact lenses, haemostatic bandages, and biosensors.^4^ Nerves are soft tissues, so materials that promote the growth and differentiation of nerve cells should be relatively soft to better simulate the microenvironment around nerves. This also makes hydrogel an ideal choice for nerve tissue engineering. The inherent three‐dimensional porous structure of hydrogel can help cells absorb, proliferate and migrate in vivo, and this three‐dimensional structure is conducive to the transport and storage of nutrients and growth factors. This also makes hydrogel an ideal choice for nerve tissue engineering. At present, conductive hydrogels commonly used in tissue engineering are usually copolymerized mixtures of conductive polymers (CPs) and non‐conductive hydrogels.^20^ Due to the existence of non‐conductive hydrogel matrix, this conductive hydrogel is easy to lead to the filtration of CP components under physiological conditions, which leads to the decrease of its conductivity and increased toxicity. It is reported that through the interaction between tannic acid (TA) and polypyrrole (PPy), a porous, highly conductive, self‐assembled conductive hydrogel without insulating polymer can be prepared.^21^ Although the hydrogel has high conductivity, and the ability to promote the differentiation of neural stem cells, TA‐PPy conductive hydrogels exhibit poor cellular performance due to the lack of bioactive domains, which play an important role in inducing cell adhesion, diffusion, and neurite extension. RGD peptide is composed of three amino acids: L‐arginine, glycine, and L‐aspartate. As an integrin ligand, it is not only the main anchoring molecule, but also plays an important role in embryogenesis, cell differentiation, immune response, wound healing, and haemostasis.^22^ Therefore, it is widely used in tissue engineering. The surface modification of TA‐PPY conductive hydrogel with RGD peptide can endow the conductive hydrogel with biological activity, which is expected to further enhance its ability to promote nerve regeneration. In recent years, polylactic acid co caprolactone (PLCL) has been proven to be a potential and effective candidate conduit material for nerve regeneration due to its biocompatibility, biodegradability, and good mechanical properties. Studies have shown that compared to single synthesized PLCL neural conduits, PLCL/SF neural conduits have a larger porosity and higher biocompatibility.^23^ In addition, adding nerve growth factor (NGF) to the neural conduit can further promote nerve regeneration and improve stent performance. The PLCL/SF/NGF@TA‐PPy‐RGD prepared in this study can release NGF, and conductive gel modifies RGD can provide anchor points for nerve cells. Thus providing a good environment for nerve regeneration, PLCL/SF/NGF@TA‐PPy‐RGD not only promotes the proliferation of nerve cells, but also promotes the generation of blood vessels around the nerve, thus promoting the regeneration and functional recovery of peripheral nerves.

Hydrogen peroxide (H_2_O_2_) treatment of PC12 cells is a commonly used experimental model to study oxidative stress‐induced neuronal damage. H_2_O_2_ oxidative stress can mimic the cellular environment of nerve injury, making H_2_O_2_ treatment a relevant model for studying the mechanisms underlying peripheral nerve defects. When PC12 cells are exposed to H_2_O_2_, they undergo oxidative damage, leading to cell death and dysfunction, which closely resembles the pathological conditions observed in peripheral nerve injuries. In this study, we utilized H_2_O_2_ treated PC12 cells to simulate the oxidative stress conditions experienced by peripheral nerves following injury. This model enabled us to evaluate the neuroprotective effects of the synthesized conductive gel TA‐PPy‐RGD and the PLCL/SF nerve conduit loaded with NGF.

The purpose of this study is to construct the PLCL/SF/NGF@TA‐PPy‐RGD, which can release NGF to promote the proliferation of nerve cells, while the conductive gel modified with RGD also can provides anchor points for nerve cells, thus providing a favourable environment for nerve regeneration. In vitro experiments were performed to test the effect of PLCL/SF/NGF@TA‐PPy‐RGD on the proliferation and migration of H_2_O_2_ treated PC12 cells and the effect on the PI3K/AKT signal pathway of PC12 cells. Subsequently, therapeutic effect of PLCL/SF/NGF@TA‐PPy‐RGD was studied on a rat model of sciatic nerve defect, this study can provide a theoretical basis and new strategies for the research and treatment of peripheral nerve defect.

## 2 MATERIALS AND METHODS

### 2.1 Preparation of PLCL/SF and PLCL/SF/NGF nanofiber films

PLCL and silk fibroin (SF) (PLCL: SF = 80:20, w/w (%)) were dissolved in hexafluoroisopropanol (HFIP) to prepare 8% spinning solution, for PLCL/SF/NGF nanofiber films, NGF was added to the spinning solution with a concentration of 1 μg/mL, then stirred the solution at 4°C overnight. 5 × 5 cm^2^ aluminium foil was used as the receiving device, and the distance between the aluminium foil and the needle is 10 cm. The prepared spinning solution was injected into a 10 mL syringe, connected an 18G needle to injection pump. Then, connected a high‐voltage static electricity of about 12 kV for electrospinning. The injection pump has a pushing rate of 0.4 mL/h. The collected nanofiber membranes were crosslinked with ethanol vapour for 24 h, and then dried in a vacuum dryer for 24 h. All electrospinning processes were carried out at 25°C and relative humidity of 50 ± 2%, obtained PLCL/SF nanofiber membrane was placed in a vacuum drying oven and treated at room temperature for 12 h to remove remaining solvents.

### 2.2 Preparation of TA‐PPy and TA‐PPy‐RGD conductive hydrogels

TA crosslinked and doped PPy conductive hydrogels (TA‐PPy) were prepared according to the following steps. Dissolved a certain amount of TA in H2O, prepared TA solutions with concentrations of 0.6%, and then added pyrrole (Py) with final concentrations of 0.8 mol/L to form solution A. For preparation of TA‐PPy‐RGD conductive hydrogels, EDC and NHS were used to react with RGD polypeptide and TA to prepare RGD‐TA, then RGD‐TA solution with concentration of 0.6% is added, and pyrrole (Py) with final concentration of 0.8 mol/L was added respectively to form solution A. Subsequently, dissolve a certain amount of FeCl3·6H_2_O to prepare 1.84 mol/L FeCl_3_ solution to form solution B. Put solution A and solution B into a 4°C refrigerator to cool down for 12 h, and then quickly mixed the two solutions by vortex oscillation. After continuing to react at 4°C for 12 h, take the formed conductive hydrogels out of the mould. The TA‐PPy and TA‐PPy‐RGD conductive hydrogels were obtained by soaking in a large amount of pure water to remove various water‐soluble by‐products remaining in the reaction.

### 2.3 Preparation of PLCL/SF@TA‐PPy and PLCL/SF/NGF@TA‐PPy‐RGD nerve conduit

A polytetrafluoroethylene (PTFE) rod shaped mould with a diameter of 2 mm and a length of 5 cm was used as a receiving device in the preparation of the nanofiber neural conduit shell. PLCL/SF or PLCL/SF/NGF nanofibers were uniformly spun on the surface of the PTEF mould. The thickness should be controlled at approximately 1 mm. After spinning, it should be placed in a vacuum drying oven and treated at room temperature for 12 h to remove any non‐volatile solvents. Then, the obtained PLCL/SF nanofiber nerve conduit should be removed for later use. Cut the sheath of the nerve catheter into 20 mm, use a PTFE rod mould with a diameter of 2 mm to extend into the nerve catheter for 5 mm, and injected TA‐PPy or TA‐PPy‐RGD conductive gel into the sheath of the catheter at the other end, and leaving a 3 mm hollow area at the end. After taking off the catheter, carefully cut it with an ophthalmic scissors, so that 2 mm hollow areas at both ends of the nerve catheter are used to cut the suture site of the nerve.

### 2.4 Characterization

Scanning electron microscope (SEM): the morphology of nanofiber film and conductive gel was observed and photographed with SEM (Hitachi, Japan) under 10 kV acceleration voltage.

Fourier Transform Infrared Spectrometer (FTIR): The FTIR spectrum of nanofiber film and conductive gel was recorded at room temperature using the Nicolet 6700 FITR spectrometer (Thermo Fisher of the United States) in the ATR attenuated total reflection mode. The average of each spectrum after 64 scans was 1 cm^−1^, and the collected spectrum was subtracted from the background. Export the data in Excel format and use Origin 8.0 software for drawing.

Mechanical performance of PLCL/SF and PLCL/SF/NGF: Before the performance of mechanical testing and characterization, nanofiber membrane should be equilibrated under constant temperature and humidity (ambient temperature 20°C, relative humidity 65%) for 24 h, first, cut the nanofiber membrane into 50 mm × 10 mm spline shape, and the thickness of each sample was measured using a spiral micrometre. Each sample was measured at three different locations, and the average value was taken as the final thickness. Then, fixed the nanofiber membrane in the form of strips on the material mechanics testing machine separately 1 cm above and 1 cm below the sample are used as fixed parts, with a testing length of 3 cm. The mechanical properties of the sample are tested to obtain a force tensile curve. Each sample was tested five times under the following conditions: the initial spacing between the test clamps is maintained at a length of 30 mm, the stretching speed is 10 mm/min, and the instrument accuracy is 0.001 N. Finally, the corresponding fracture strength and elongation at break for each sample are calculated using the formula.

Test the stress–strain curve and calculate the Young's modulus value.

Stress (MPa) = Breaking strength (N)/cross‐sectional area of the sample (mm^2^).

Strain (%) = (Fracture length (mm)−Initial length of sample (mm))/Initial length of sample (mm) × 100%.

Young's modulus (MPa) = stress at 10% strain (MPa)/10% (%) × 100%.

The relevant test data obtained was exported in Excel format and plotted using Origin 8.0 software.

Test of TA‐PPy‐RGD conductive gel electrical conductivity: characterize TA‐PPy and TA‐PPy‐RGD by electrochemical workstation cyclic voltammetry to test the electrical conductivity of conductive hydrogel, evenly coat 100 μL pre gel solution with different components on the conductive glass substrate (standard electrode potential (vsREH): 0.05–1.25, reference potential (vsREF saturated calomel): −0.50147‐0.6985), and seal it in an incubator at 37°C for 24 h, Select 1 mol/LKcl electrolyte solution. In the test, the scanning potential parameter was set to −0.8–1.0 V and the scanning rate was 10 mV/s.

Swelling performance test of nerve tube: the swelling coefficient is used to evaluate the swelling performance of gel. Take a number of dried gel, then soak the gel in 0.1 mol/L PBS (pH 7.2–7.4) buffer solution and incubate it in a 37°C incubator for 24 h to make the gel fully swollen, use filter paper to absorb the water on the surface of the catheter, weigh again, and calculate the swelling coefficient (SI) of the sample according to the following formula: SI (%) = (Wwet−Wdry)/Wdry × 100%, Wdry and Wwet represent the mass of the catheter before and after swelling, respectively.

### 2.5 Cell culture

Neural cell PC12 were provided by BEENbio (BEENbio, China), and cell culture was carried out using high glucose DMEM containing 10% FBS and 500 IU/mL of penicillin and 500 IU/mL streptomycin. Cells were cultured in a 37°C cell culture incubator containing 5% CO_2_. For establishing a hydrogen peroxide cell damage model, cells in the logarithmic growth phase was treated with 150 μmol/L of H_2_O_2_ for 24 h.

### 2.6 Cell viability and proliferation

Cell viability test: Each group pretreated PC12 cells was inoculate with a density of 1 × 10^4^/well into a 96 well plate, after incubated in 37°C for 24 or 48 h, add 10 μL of 5 mg/mL of CCK‐8 solution to each well. Incubate in the dark for 4 h, mix gently at room temperature for 10 min, measure the OD value at 450 nm, and calculate the cell viability rate.

Cell proliferation detection: was Inoculate with a density of 1 × 10^4^/well into a 6‐well plate, after incubated in 37°C for 0, 2, 4, 6, 8, 10, 12, and 14 h, the cell proliferation curve was plotted after cell counting.

### 2.7 PLCL/SF/NGF@TA‐PPy‐RGD release performance

ELISA kit was used to test the amount of NGF in the solution before and after loading, the prepared PLCL/SF/NGF@TA‐PPy‐RGD was placed in 4 mL PBS (pH 7.4), and then placed in dialysis bags. The dialysis bags were placed in 6 mL PBS and stirred at low speed at 37°C. At each sampling point, 4 mL of the solution on the outside of the dialysis bag was taken and the concentration was measured using an NGF ELISA kit. At the same time, an equal volume of solution was added for subsequent testing.

### 2.8 Staining of live and dead cells

Inoculated PC12 cells into 96 well plates, and treated the cells according to the experimental design. Removed the culture medium, washed the cells once with PBS, and added an appropriate volume of Calcein AM/PI detection working fluid. Incubated at 37°C in dark for 30 min. After incubation, observed the cells under a fluorescence microscope and image.

### 2.9 Cell clone formation experiment

After 24 h of pre‐treatment with PLCL/SF or PLCL/SF/NGF and TA‐PPy or TA‐PPy‐RGD, PC12 cells were rinsed with PBS and inoculated into sterile 6‐well plates at a rate of 500–800 cells per well. Then, 2 mL of cell culture medium was added, followed by blowing with a l mL gun until uniform. The cells were placed in a cell culture incubator for cultivation, and half of the culture medium was changed every 3 days. After 2 weeks, discard the culture medium, gently rinse once with PBS, fix the experimental group cells with methanol for 10 min, and further stain with crystal violet for 5 min. Finally, scan the image and save it.

### 2.10 Cell scratch testing

PC12 cells were cultured in a 6 cm culture dish. After 24 h of pre‐treatment with PLCL/SF or PLCL/SF/NGF and TA‐PPy or TA‐PPy‐RGD, PC12 cells were rinsed with PBS and continued to be cultured. After the cells grew to 80%–90%, a straight line was drawn in the centre of each well. Discard the supernatant and wash twice with PBS. After each well treatment, culture for 48 h and take photos under an inverted fluorescence microscope to observe the remaining distance of cells in each group after migrating from the edge of the scratch to the centre of the scratch.

### 2.11 Transwell assay

PC12 cells were cultured in a 6 cm culture dish, After 24 h of pre‐treatment with PLCL/SF or PLCL/SF/NGF and TA‐PPy or TA‐PPy‐RGD, PC12 was rinsed with PBS and continue to culture. After the cells grow to 80%–90%, adjust the cell concentration to 1.0 × 10^5^/mL with serum‐free medium and then cells were inoculated into the upper chamber of transwell with an amount of 100 μL/well. After treatment, the cells in each group were further cultured at 37°C for 24 h. Remove the upper chamber of transwell chamber, wiped off the cells on the surface of the polycarbonate membrane with a cotton swab, washed twice with PBS, and fixed the upper chamber in 4% paraformaldehyde for 15 min. after washed with PBS twice, stain with haematoxylin for 20 min, then washed with PBS twice. Count the transmembrane cells from five different fields under an inverted fluorescence microscope and calculated the average value.

### 2.12 Haemolytic experiment

Before the experiment, 10 mm × 30 mm PLCL/SF and 0.1 g TA PPy gel were soaked in 75% ethanol for 0.5 h for sterilization, and then washed three times with sterile water. Firstly, healthy red blood cells (HRBCs) were obtained from fresh rabbit blood containing 3.8% sodium citrate injection (sodium citrate to distilled water ratio, 3.8:100 w/v). Centrifuge fresh blood (1200 rpm, 10 min) and completely remove the serum to obtain HRBCs. Wash the precipitate five times with PBS. Dilute healthy HRBCs 10 times with PBS before the haemolysis experiment. Mix the diluted HRBCs (0.2 mL) with PBS solution (10 mL) and add them to a centrifuge tube, with a total volume of 10.2 mL. Diluted HRBCs (0.2 mL) were mixed with 10 mL of water and 10 mL of PBS buffer as positive and negative controls, respectively. After gentle vibration, all samples were incubated at 37°C for 2 h, and then centrifuged at 2000 rpm for 5 min. Carefully remove the clarified liquid from the upper layer. The supernatant was tested for absorbance at 545 nm using a UV visible spectrophotometer.

### 2.13 Acute toxicity detection of neural conduit in mice

Twenty healthy male Balb‐C mice, 18–20 g, were selected and divided into groups of 10 mice per group. The mice were given food and water without interruption the night before the pre experiment, After PLCL/SF or TA‐PPy intraperitoneal, observe the mental state, diet, non‐toxic reactions, and mortality of mice. After 3 days of observation, dissected and observed the organs of mice in the drug group for any abnormal lesions. The purpose of the pre experiment is to measure the LD50 of mice in the drug group. If any mice die in the pre experiment, the formal experiment will determine the LD50 by intraperitoneal at different concentrations and times based on the number of dead mice in the pre experiment. If there were no mouse deaths in the preliminary experiment, the sample size should be increased for the main experiment. In the main experiment, as no mice died during the preliminary phase, 45 healthy male mice weighing between 18 and 20 g were selected. These mice were divided into three groups, with 15 mice per group. On the night before the experiment, the mice were fasted but allowed unrestricted access to water. On the day of the experiment, the mice received treatment with either PBS, PLCL/SF, or TA‐PPy. Their condition was monitored post‐intraperitoneal injection, with observations and records of their weight, mental state, toxic reactions, and mortality made every other day. At the conclusion of the experiment, the mice were anaesthetised, and their hearts, livers, spleens, lungs, and kidneys were harvested for comparison among the control, PLCL/SF, and TA‐PPy groups. The changes in each organ were observed using HE staining.

### 2.14 Detecting inflammatory factors with ELISA method

Digested and collected cells, centrifuge at 1000 rpm/min, washed twice with PBS, treated cells with RIPA cell lysate for 10 min, centrifuge at 12000 rpm/min, collected supernatant, and store at −20°C. Follow the instructions of the R&D system ELISA kit, and taken 50 μL the serum and standard substance in a 96 well plate, then add 50 μL diluent, incubated at room temperature for 2 h, discarded the liquid, add washing solution and repeat washing three times, added 100 μL to each well enzyme‐linked immunosorbent assay was used to detect antibodies. Incubated at room temperature for 2 h, discarded the liquid, added washing solution, and repeat washing three times. Added 100 μL to each well colour solution, incubated at room temperature in dark for 30 min, added 100 μL stop solution, mixed thoroughly to terminate the reaction, and measured the OD value at 450 nm on a full function enzyme‐linked immunosorbent assay (ELISA) reader. Drawn standard curves based on different concentrations of standard substances and their corresponding OD values, and obtain the concentration of IL‐6, IL‐10 and TNF‐α.

### 2.15 H&E staining

After the mouse administration experiment, 10% chloral hydrate was used for anaesthesia, and organ tissues were taken after euthanasia. Fix in 10% formalin for 24 h, wash with water for 12 h, embed in dehydrated paraffin, and slice for about 6 μm. Place the slices in a 60°C oven for 30 min to dry for later use. Soak the dried tissue slices in xylene I and xylene II for 5 min each for dewaxing, and immerse the dewaxed tissue slices in 100%, 95%, 80% and 70% alcohol for 2 min each. wash twice for 1 min. Stain with haematoxylin for 5 min, rinse with tap water for 3 s, colour separation in acidic alcohol with 1% hydrochloric acid for 2 s, rinse with tap water for 2 min, then immerse in 95% alcohol and 100% alcohol separately and dehydrate twice, each time for 2 min. Soak in eosin for 5 s and rinse with tap water for 3 min. Then immerse the tissue slices in 95% alcohol and 95% alcohol for 3 min, and finally immerse them in xylene I and xylene II for 5 min. Neutral resin seal, microscopic inspection.

### 2.16 Flow cytometry detection of cell apoptosis

When established a H_2_O_2_ cell damage model, cells in the logarithmic growth phase was treated with 150 μmol/L of H_2_O_2_ for 24 h. Cell apoptosis was detected using flow cytometry and Annexin V‐FITC/PI apoptosis detection kit, PLCL/SF/NGF and TA‐PPy‐RGD Inoculate PC12 in the logarithmic growth phase into a 12 well plate and perform pre‐treatment according to the experimental design. Digest the cells and washed twice with PBS, and resuspend with PBS. Take 50,000–100,000 resuspend cells, centrifuge 1000*g* for 5 min, discarded the supernatant, and added 195 μL. Gently resuspend cells with Annexin V‐FITC binding solution. Join 5 μL Annexin V‐FITC, gently mixed well. Join 10 μL mixed the PI staining solution. Incubated at room temperature (20–25°C) in dark for 10–20 min, then place in an ice bath. During the incubation process, cells can be resuspended 2–3 times to improve staining efficiency. Immediately perform flow cytometry analysis on the machine. Draw a bar chart based on the ratio of apoptotic cells.

### 2.17 Construction of a rat animal model with sciatic nerve defect

Adult male SD rats were used as experimental animals, 240–280 g. They were raised during the day and night period for 12 h, with a room temperature of 26°C and free drinking water. Ten rats were selected from each group, with the right hind limb of the rat as the experimental side and the left hind limb as the control side. Before the surgery begins, soak the outer shell of the nerve conduit in PBS buffer containing dual antibody solution (penicillin 500 IU/mL, streptomycin 500 IU/mL) for 30 min. Brush all surgical instruments with flowing water until clean and stain free, then wiped with alcohol cotton balls and sterilize with high‐pressure steam. After wiping the body position board with alcohol, place it on the operating table and irradiate it with a UV lamp for 30 min. After weighing the rats, they were anaesthetised by intraperitoneal injection of 1% pentobarbital sodium solution (40 mg/kg). They were placed in a prone position and fixed on a position plate. The surgical side of the hind limbs was subjected to hair removal and skin preparation treatment at the surgical site. After exposing the skin, the surgical area was wiped and disinfected with a 75% alcohol cotton ball, and a sterilized surgical cavity towel was applied to fully expose the surgical skin. After taking the thigh, an oblique incision was made to cut open the skin and separate the connective tissue between the skin and muscles. Surgical scissors are used to bluntly separate the dorsal gluteus muscle along the direction of the muscle fibres, exposing the sciatic nerve. Then operate under a 10× magnification surgical binocular microscope: use microsurgical instruments to peel off the connective tissue between the nerve and muscle tissue, fully free the sciatic nerve, use microsurgical scissors to cut the nerve 5 mm below the piriformis muscle, and removed 10 mm of nerve tissue to the distal end, creating a nerve defect model. The experimental group used different materials of catheters to suture the distal and proximal ends of the nerve, and sutured the outer membrane of the nerve to the outer wall of the neural conduit using 8–0 non absorbable suture thread; the control group will invert the severed nerve tissue and perform end‐to‐end anastomosis with the distal and proximal ends of the nerve. All anastomoses were sutured with three stitches. Suture the muscle layer and skin layer of the wound layer by layer using 4–0 surgical sutures. Intraperitoneal injection of 1 mL of dual antibody solution (penicillin, streptomycin 105 IU/kg) to prevent postoperative infection. After surgery, all experimental rats were grouped and raised in cages, labelled, freely fed and drank, and their general condition and wound healing were observed daily.

### 2.18 qPCR assay

PC12 in the logarithmic growth phase was inoculated into a 12 well plate and subjected to pre‐treatment according to the experimental design of each group. The cells were digested with trypsin, washed twice with PBS, resuspended with PBS, and digested with trypsin. After washed with PBS for two times, 1 mL of trizol reagent was added. Added 1 mL of trizol to the extracted cells for complete lysis, repeatedly blow and mix, and leave at room temperature for 5 min; 200 μL of chloroform, covered with a lid, vigorously shake for 15–30 s, and let it sit at room temperature for 2–15 min; After thorough mixing, 4°C × Centrifuge at 12000 rpm for 20 min, transferred the upper clear liquid to a new EP tube, added 500 uL of isopropanol, shake well, and leave at room temperature for 15 min; Added 1 mL of 75% ethanol to wash and precipitate twice at 4°C × Centrifuge at 12000 rpm for 5 min, discarded the supernatant, let it sit at room temperature for 5–10 min, air dry, added 20 μL of RNase Free H_2_O_2_, and fully dissolved RNA; Using a UV spectrophotometer (ND‐1000, Nanodrop Technologies) to identify RNA concentration and purity, the OD = 260/280 ratio is often between 1.8 and 2.0. Calculate the required RNA volume based on RNA concentration. Reverse transcription kit was used to reverse mRNA into cDNA, and then SYBR Green qPCR detection kit was used to detect RNA expression levels in each group. PCR cycle parameters: 95°C, 10 min, 95°C, 10 s, 60°C, 1 h, a total of 40 cycles, extended at 72°C for 7 min.

### 2.19 Western blot

PC12, which was in the logarithmic growth phase, was inoculated into a 12 well plate and subjected to pre‐treatment according to the experimental design of each group. The cells were digested with trypsin, washed twice with PBS, centrifuged at 1500 rpm for 5 min, and cell precipitates were collected. 500 μL of RIPA cell lysate was added, and the cells were lysed at 4°C for half an hour. Using BCA protein quantification method to detect protein concentration, extract 80 μL of protein and add 5 × add 20 μL of sample buffer, heat at 100°C or boiling water bath for 10 min to fully denature the protein; 10% SDS‐PAGE gel can be used to sample and separate proteins. The low voltage can be set at 80–100 V, and the high voltage can be set at about 120 V. The PVDF membrane was immersed in methanol for 5 min, and the SDS‐PAGE gel and PVDF membrane were assembled and then converted to 100 V for 2 h. After the membrane transfer is completed, immediately place the membrane in a pre‐prepared western sealing solution (5% skim milk), seal for 1 h, and dilute the primary antibody (BCL‐2, Bax, Caspase‐3, PI3K, p‐PI3K, AKT, p‐AKT, Erk, p‐Erk, NF‐200, MBP, CD34, VEGF, GAPDH) with 5% skim milk in an appropriate proportion using western sealing solution. Slowly shake the primary antibody on a side shaking table at room temperature for 1 h, post recovery of primary antibody; wash the film with TBST four times, each time for 10 min; dilute the secondary antibody with 5% skimmed milk in western blocking solution in an appropriate proportion (1:1000), and incubate the secondary antibody for 1 h; Wash the film with TBST four times, each time for 10 min; after absorbing moisture from the film, use SuperSignal West Pico chemiluminescence substrate to emit light and colour, and expose the film.

### 2.20 Rat footprint analysis experiment and SFI index evaluation and gastrocnemius muscle weighing

At 12 weeks post‐surgery, the sciatic nerve function index (SFI) was used to evaluate the postoperative foot motor function of rats. The procedure is as follows: construct a narrow walking passage with a length of 80 cm and a width of 10 cm using cardboard. One end of the passage is the entrance, and the other end is connected to a black wooden box. Place white paper at the bottom of the passage to record rat footprints. Wet both sides of the hind feet of the experimental rat with a cotton ball dipped in black ink, and then place it at the entrance of the walking passage. After it crawls forward into the black box, about 4–5 footprints are left on each side of the white paper. Select clear and distinguishable footprints on both sides for measurement. The length from heel to toe is the paw length (PL), and the distance between the first and fifth toes is the toe opening distance (Toe spread, TS), The distance between the second and fourth toes is the Intermediate toe spread (IT), with E and N prefixes representing the experimental and control side data, respectively. These values are applied to the Bain formula to calculate SFI. When the SFI value is 0, it indicates complete normal sciatic nerve function, and −100 indicates complete loss of sciatic nerve function.

Bain formula:

$$\mathrm{SFI}=-38.3\times \left[\left(\mathrm{EPL}-\mathrm{NPL}\right)/\mathrm{NPL}\right]+109.5\times \left[\left(\mathrm{ETS}-\mathrm{NTS}\right)/\mathrm{NTS}\right]+13.3\times \left[\left(\mathrm{EIT}-\mathrm{NIT}\right)/\mathrm{NIT}\right]-8.8$$

Dissect the gastrocnemius muscle from each group of rats and weigh it.

### 2.21 Immunofluorescence detection of damaged nerves

Soak the dried tissue sections in xylene I and xylene II for 5 min each for dewaxing, and immersed the dewaxed tissue sections in 100%, 95%, 80% and 70% alcohol for 2 min each. Wash three times with PBS, permeated 0.2% Triton X‐100 for 30 min, washed three times with PBS, aspirated the liquid, and sealed with goat serum (or 2% BSA‐PBS solution) at room temperature for 30 min. Removed the blocking solution, diluted the primary antibody with PBS containing 2% BSA (dilution ratio 1:50–1:500), incubated the cells in a wet box, overnight at 4°C, removed the primary antibody, washed three times with PBS, diluted the Cy3 labelled fluorescent secondary antibody with PBS containing 2% BSA (dilution ratio 1:500), incubated at room temperature for 1 h, removed the fluorescent secondary antibody, and washed three times with PBS. Washed with PBS three times, then stained the cell nucleus with 0.01% DAPI staining solution for 5 min. Washed with PBS three times, take a clean cover glass, and use 3 μL of anti‐fluorescence quenching sealing solution is used to seal the film, with the tissue facing upwards to avoid bubbles and absorb excess sealing agent. The film is then observed and photographed under a laser confocal microscope.

### 2.22 Masson staining

The tissue fixed with 4% paraformaldehyde, dehydrated step by step with ethanol, embedded in conventional paraffin, paraffin sections were dewaxed to water, stained with Wiegert haematoxylin for 10 min, stained with acid fuchsin for 10 min, washed with 0.2% acetic acid aqueous solution for 5 s, treated with 1% phosphomolybdic acid aqueous solution for 5 min, washed with 0.2% acetic acid aqueous solution for 5 s, stained with aniline blue solution for 5 min, washed with 0.2% acetic acid aqueous solution for 5 s, dehydrated with 95% ethanol and anhydrous ethanol, and transparent with xylene, neutral resin sealing. Finally, observe the slices under a microscope and take photos.

### 2.23 Data statistics

Statistical analyses were processed using GraphPad Prism 9.0 and SPSS version 26.0 software. To compare the means between the experimental and control groups, an independent t‐test was used for normally distributed data, while a Mann–Whitney *U* test was applied for non‐normally distributed data. All of the results were presented as the mean value plus a standard deviation (±SD) from at least three independent experiments. For comparisons involving more than two groups, one‐way ANOVA followed by Tukey's HSD post hoc test was used for normally distributed data. For non‐normally distributed data, Kruskal–Wallis test followed by Dunn's post hoc test was applied. *p ≤ 0.05, **p ≤ 0.01, ***p ≤ 0.001 were considered statistically significant.

## 3 RESULTS

### 3.1 Preparation and characterization of RGD modified TA‐PPY conductive hydrogel

**FIGURE 1:**
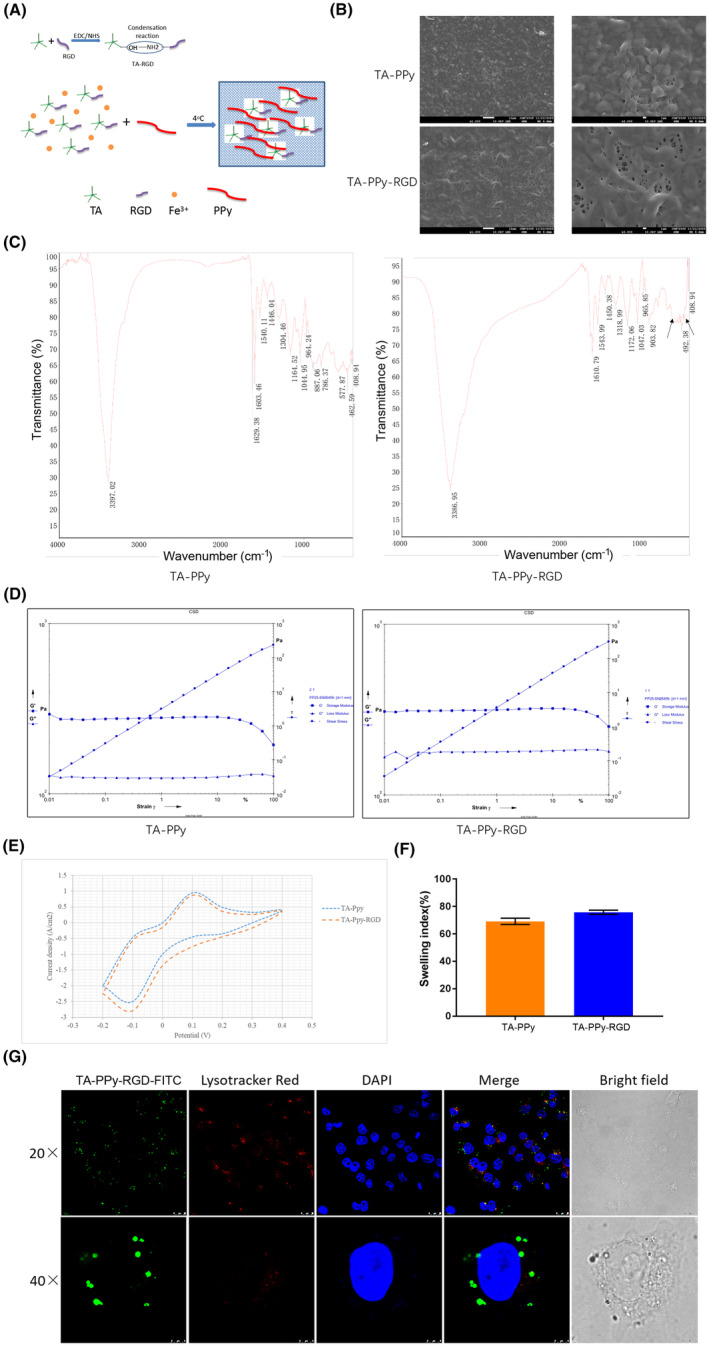
Preparation and characterization of RGD modified TA‐PPY conductive hydrogel. (A) The model of preparation of RGD modified TA‐PPY conductive hydrogel, (B) the SEM images of TA‐PPy and TA‐PPy‐RGD, (C) FTIR spectra of TA‐PPy and TA‐PPy‐RGD, (D) the mechanical of the TA‐PPy and TA‐PPy‐RGD, the storage modulus (G′) and loss modulus (G) of TA‐PPy and TA‐PPy‐RGD were measured by rheological method, (E) The electrochemical properties of TA‐PPy and TA‐PPy‐RGD were detected by cyclic voltammetry (CV). (F) The swelling performance of TA‐PPy and TA‐PPy‐RGD, (G) laser confocal microscope was used to prove the TA‐PPy‐RGD can effectively combine with the surface of nerve cell membrane to provide growth anchor site for never cells. PPy, polypyrrole; TA, tannic acid.

Firstly, according to the experimental process shown in Figure 1A, RGD modified TA‐PPy conductive hydrogel was synthesized, the SEM was used to characterize the crystalline structure and morphology of the TA‐PPy and TA‐PPy‐RGD. As show in Figure 1B, both TA‐PPy and TA‐PPy‐RGD have a 3D microporous foam network. Compared with TA‐PPy, TA‐PPy‐RGD has larger microporous structure which can provide larger effective surface area for material exchange and cell adhesion. FTIR spectra of TA‐PPy and TA‐PPy‐RGD were shown in Figure 1C. Results showed that RGD was successfully modified on the TA‐PPy, and the perks of C‐N combined with RGD was observed around 492.38 cm^−1^. The storage modulus (G′) and loss modulus (G) of TA‐PPy and TA‐PPy‐RGD were measured by rheological methods, as shown in the Figure 1D, TA‐PPy and TA‐PPy‐RGD have similar storage modulus (G′), but TA‐PPy‐RGD has lower energy dissipation modulus than TA‐PPy which suggests that both of them have the same elasticity, while TA‐PPy‐RGD has better crosslinking performance. The electrochemical properties of TA‐PPy and TA‐PPy‐RGD were detected by cyclic voltammetry (CV). The results showed that both TA‐PPy and TA‐PPy‐RGD has good current response between −0.2 and 0.4 V (Figure 1E), The measurement of swelling coefficient was used to evaluate the swelling performance of the TA‐PPy and TA‐PPy‐RGD. The results showed that the swelling coefficients of TA‐PPy and TA‐PPy‐RGD were <80% (Figure 1F), and there was no significant difference between the TA‐PPy and TA‐PPy‐RGD. The aim of RGD modification on the TA‐PPy conductive hydrogel was provide a growth anchor site for nerve cells. As shown in the Figure 1G, FITC labelled TA‐PPy‐RGD was incubated with PC12 cell for 24 h, obvious green fluorescence can be observed around the cells, which indicated that the TA‐PPy‐RGD can effectively combine with the surface of nerve cell membrane to provide growth anchor site.

### 3.2 Preparation and characterization of PLCL/SF and PLCL/SF/NGF nanofiber film

**FIGURE 2:**
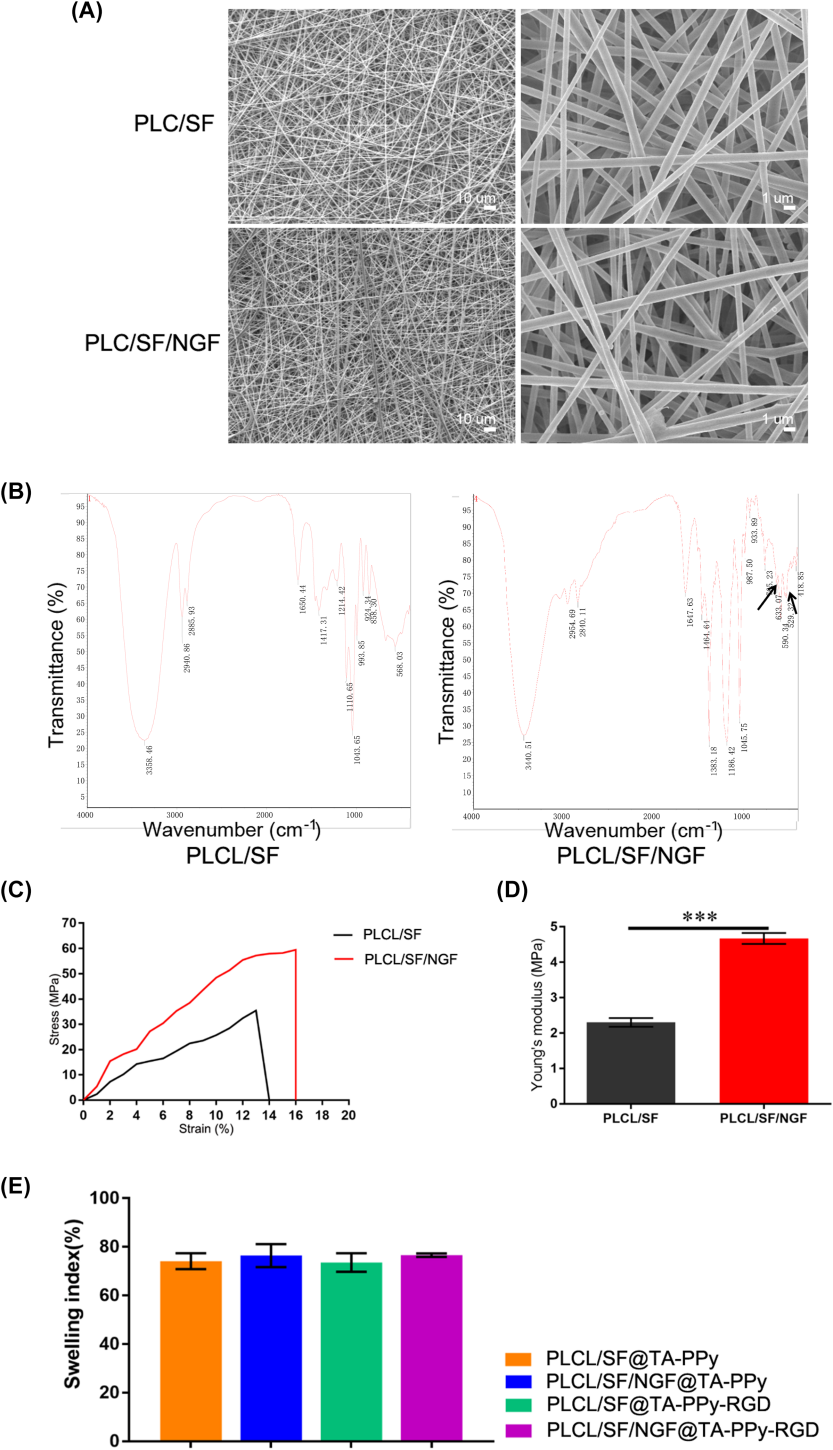
Preparation and characterization of PLCL/SF and PLCL/SF/NGF nanofiber film. (A) The SEM image of PLCL/SF and PLCL/SF/NGF nanofiber film, (B) the FTIR spectra of PLCL/SF and PLCL/SF/NGF nanofiber film, (C) the mechanical properties of PLCL/SF and PLCL/SF/NGF nanofiber film, (D) the tensile strength and Young's modulus of PLCL/SF and PLCL/SF/NGF nanofiber film, (E) after we injected the TA‐PPy or TA‐PPy‐RGD into the PLCL/SF and or PLCL/SF/NGF shell and constructed the different nerve conduit was constructed, and the swelling performance was tested, as showed in the (E), the constructed nerve conduit had good swelling performance which reaching the standard of clinical nerve conduit. NGF, nerve growth factor; PLCL, polylactic acid co caprolactone; PPy, polypyrrole; TA, tannic acid. *p < 0.05, **p < 0.01, and ***p < 0.001.

By use of electrospinning, we prepared PLCL/SF and PLCL/SF/NGF nanofiber film was prepared here, Figure 2A shows the SEM image of PLCL/SF and PLCL/SF/NGF nanofiber films, The diameters of the synthesized PLCL/SF and PLCL/SF/NGF was 0.4–0.8 μm. Figure 2B shows the FTIR spectra of PLCL/SF and PLCL/SF/NGF nanofiber films. Subsequently, we tested the mechanical properties of PLCL/SF and PLCL/SF/NGF nanofiber films (Figure 2C). The stress–strain curve results show that the ultimate stress and strain of PLCL/SF/NGF improved compared to PLCL/SF (Figure 2C). The tensile strength and Young's modulus of PLCL/SF/NGF were 59.4 ± 1.0 MPa and 2.3 ± 0.1 MPa, respectively, which were superior to PLCL/SF, which had values of 35.5 ± 1.4 MPa and 4.67 ± 0.13 MPa (Figure 2D). Finally, we injected the TA‐PPy or TA‐PPy‐RGD into the PLCL/SF and PLCL/SF/NGF shells and constructed the nerve conduit, and the swelling performance was tested, as shown in the Figure 2E, the constructed nerve conduit had good swelling performance, reaching the standard of clinical nerve conduit.

### 3.3 Biocompatibility of TA‐PPy and PLCL/SF

**FIGURE 3:**
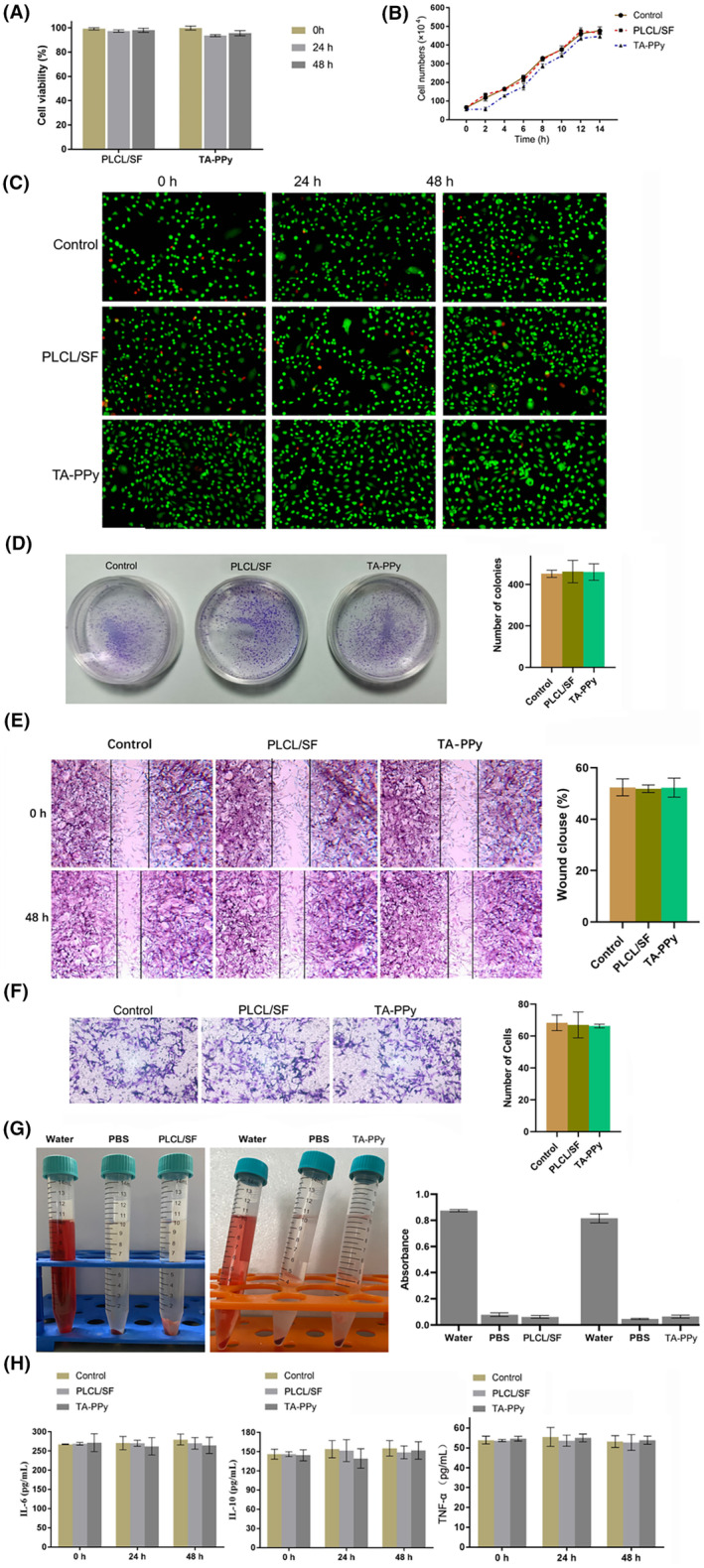
Biocompatibility of TA‐PPy and PLCL/SF. The CCK‐8 assay (A), cell growth curve (B), calcein AM/PI cell fluorescence detection kit (C) and cell clone formation assay (D) were used to test the effects of PLCL/SF and TA‐PPy on the cell viability and proliferation of PC12 cells, cell scratch (E) and transwell assay (F) were used to test the effects of PLCL/SF and TA‐PPy on the migration of PC12 cells, (G) the haemolytic performance of PLCL/SF and TA‐PPy, (H) the effects of PLCL/SF and TA‐PPy on the levels of IL‐6, IL‐10 and TNF‐α in serum of mice. PLCL, polylactic acid co caprolactone; PPy, polypyrrole; TA, tannic acid. *p < 0.05, **p < 0.01, and ***p < 0.001.

The CCK‐8 assay (Figure 3A), cell growth curve (Figure 3B), calcein AM/PI cell fluorescence detection kit (Figure 3C), and cell clone formation assay (Figure 3D) were used to test the effects of PLCL/SF and TA‐PPy on the cell viability and proliferation of PC12 cells. The results showed that PLCL/SF and TA‐PPy had no significant effect on the activity and proliferation of PC12 cells. The cell scratch assay (Figure 3E) and transwell assay (Figure 3F) were used to test the effects of PLCL/SF and TA‐PPy on the migration of PC12 cells, and the results showed that PLCL/SF and TA‐PPy had no significant effect on the migration of PC12 cells. Blood compatibility is one of the decisive factors for engineering scaffolds. The physical and chemical substances on the surface can damage red blood cells, leading to the release of haemoglobin. Studied the degree of red blood cell dissolution when PLCL/SF and TAA‐PPy were introduced into red blood cells to determine blood compatibility. The haemolysis test results are shown in Figure 3G. The absorbance of the supernatant after PLCL/SF and TAA‐PPy tests showed no difference compared to the PBS group. Subsequently, the effects of PLCL/SF and TA‐PPy on the levels of IL‐6, IL‐10 and TNF‐α in serum of mice were tested. The results showed that PLCL/SF and TA‐PPy had no significant effects on the levels of inflammatory factors in cells and serum of mice (Figure 3H).

### 3.4 Effects of PLCL/SF/NGF and TA‐PPy‐RGD on proliferation, migration, and apoptosis of H_2_O_2_ treated PC12 cells

**FIGURE 4:**
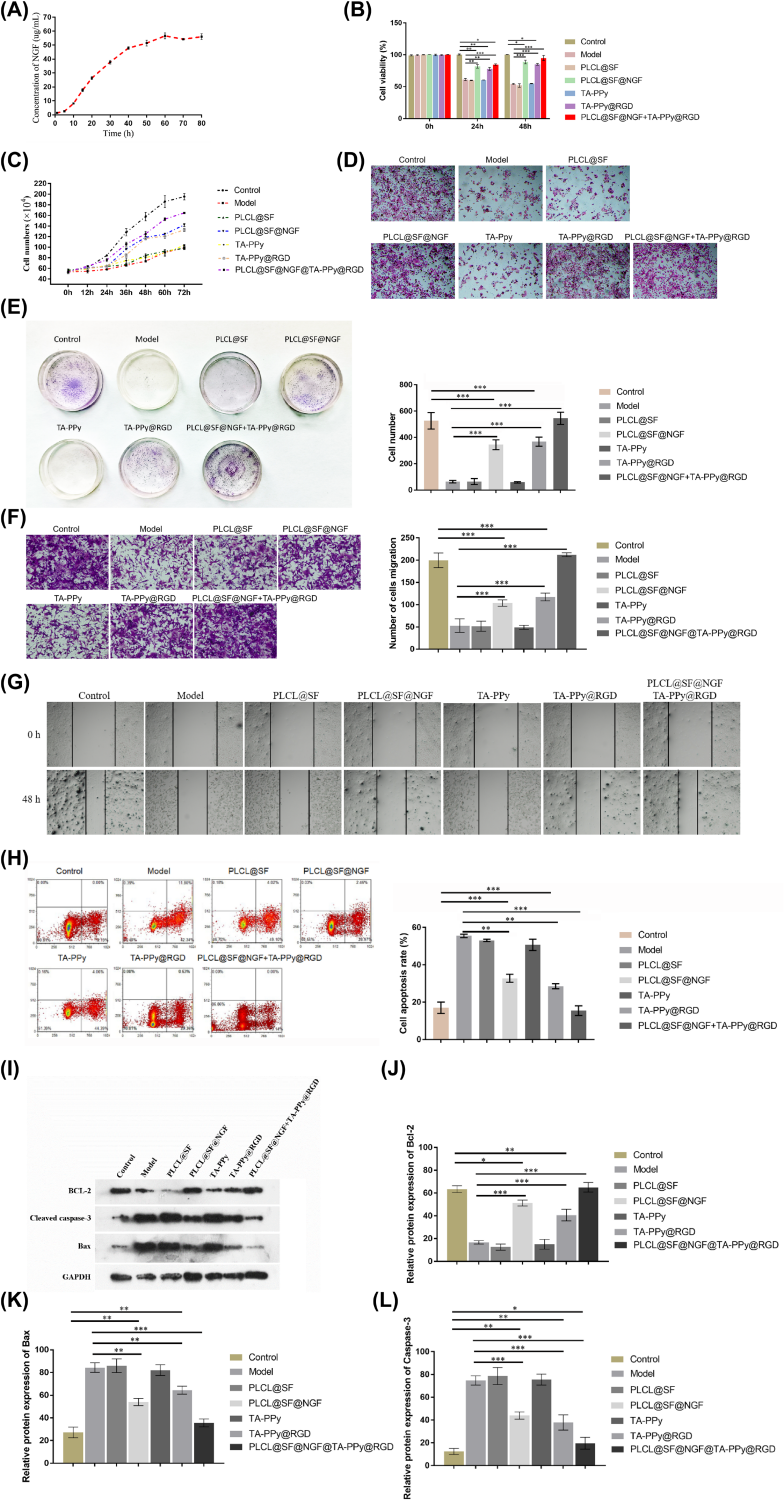
Effects of PLCL/SF/NGF and TA‐PPy‐RGD on proliferation, migration, and apoptosis of H_2_O_2_ treated PC12 cells. (A) The NGF release performance of PLCL/SF/NGF nanofiber film, CCK‐8 assay (B), cell growth curve (C) and cell clone formation assay (E) were used to evaluate the effects of PLCL/SF/NGF and TA‐PPy‐RGD on the activity and proliferation of PC 12 cells which treated with H_2_O_2_, (D) HE staining image of PC12 cells after 48 h of treatment, the cell transwell (F) and cell scratch (G) assay were used to evaluate the effects of PLCL/SF/NGF and TA‐PPy‐RGD on the migration of PC12 cells which treated with H_2_O_2_, (H) flow cytometry and Annexin V/PI apoptosis detection kit was used to test the effect of PLCL/SF/NGF and/or TA‐PPy‐RGD on the apoptosis of PC12 cells which treated with H_2_O_2_, western blot (I) and qPCR (J‐L) assay were used to detect the effects of PLCL/SF/NGF and TA‐PPy‐RGD on the levels of apoptosis related proteins (Bcl‐2, Caspase‐3, Bax) in PC12 cells treated with H_2_O_2_. NGF, nerve growth factor; PLCL, polylactic acid co caprolactone; PPy, polypyrrole; TA, tannic acid. *p < 0.05, **p < 0.01, and ***p < 0.001.

The NGF release performance of PLCL/SF/NGF nanofiber film was tested (Figure 4A). PC12 cells without treated with PBS was used as blank control, and the PC12 cells treated with 150 μM of H_2_O_2_ for 24 h was used as negative control. The results showed that the PLCL/SF/NGF nanofiber film can release NGF within 60 h and then maintain a relatively stable concentration of NGF at least 20 h. PC12 cell was treated with 150 μM of H_2_O_2_ for 24 h to constructing a neural cell oxidative damage model, then CCK‐8 assay (Figure 4B), cell growth curve (Figure 4C) and cell clone formation assay (Figure 4E) were used to evaluate the effects of PLCL/SF/NGF and TA‐PPy‐RGD on the activity and proliferation of PC12 cells which treated with H_2_O_2_. The results showed that after co‐culture with PLCL/SF/NGF and/or TA‐PPy‐RGD for 24 and 48 h, PLCL/SF/NGF and TA‐PPy‐RGD significantly increased the activity and proliferation of PC12 cells. Futhermore, PLCL/SF/NGF and TA‐PPy‐RGD had a synergistic effect. The PLCL/SF/NGF + TA‐PPy‐RGD group significantly increased the activity and proliferation of PC12 cells compared to the PLCL/SF/NGF and TA‐PPy‐RGD groups. Figure 4D showed the HE staining image of PC12 cells after 48 h of treatment. The cell transwell (Figure 4F) and cell scratch (Figure 4G) assay were used to evaluate the effects of PLCL/SF/NGF and TA‐PPy‐RGD on the migration of PC12 cells which treated with H_2_O_2_. The results showed that after co‐culture with PLCL/SF/NGF and/or TA‐PPy‐RGD for 48 h, both PLCL/SF/NGF and TA‐PPy‐RGD significantly increased PC12 migration, while PLCL/SF/NGF and TA‐PPy‐RGD had a synergistic effect. The PLCL/SF/NGF + TA‐PPy‐RGD group significantly increased PC12 migration compared to PLCL/SF/NGF and TA‐PPy‐RGD groups. The effects of PLCL/SF/NGF and TA‐PPy‐RGD on the apoptosis of H_2_O_2_ treated PC12 cells were evaluated by using flow cytometry and Annexin V/PI apoptosis detection kit, as showed in the Figure 4H, after co‐culture with PLCL/SF/NGF and/or TA‐PPy‐RGD for 48 h, both PLCL/SF/NGF and TA‐PPy‐RGD could significantly reduce the apoptosis of PC12 cell, the PLCL/SF/NGF + TA‐PPy‐RGD group significantly reduce PC12 cell apoptosis compared to the PLCL/SF/NGF and TA‐PPy‐RGD groups. Subsequently, Western blot (Figure 4I) and qPCR (Figure 4J–L) assay were used to detect the effects of PLCL/SF/NGF and TA‐PPy‐RGD on the levels of apoptosis related proteins in PC12 cells treated with H_2_O_2_. The results showed that PLCL/SF/NGF and TA‐PPy‐RGD could significantly increase the level of Bcl‐2 in PC12 cells while significantly reducing the levels of Caspase‐3 and Bax. With the PLCL/SF/NGF and TA‐PPy‐RGD had a synergistic effect, with the PLCL/SF/NGF + TA‐PPy‐RGD group showing a higher level of Bcl‐2 compared to the PLCL/SF/NGF and TA‐PPy‐RGD groups, and a significant difference in the levels of Caspase‐3 and Bax.

### 3.5 PLCL/SF/NGF and TA‐PPy‐RGD regulating the effect of H_2_O_2_ on the PC12 cell by activating the PI3K/AKT signalling pathway

**FIGURE 5:**
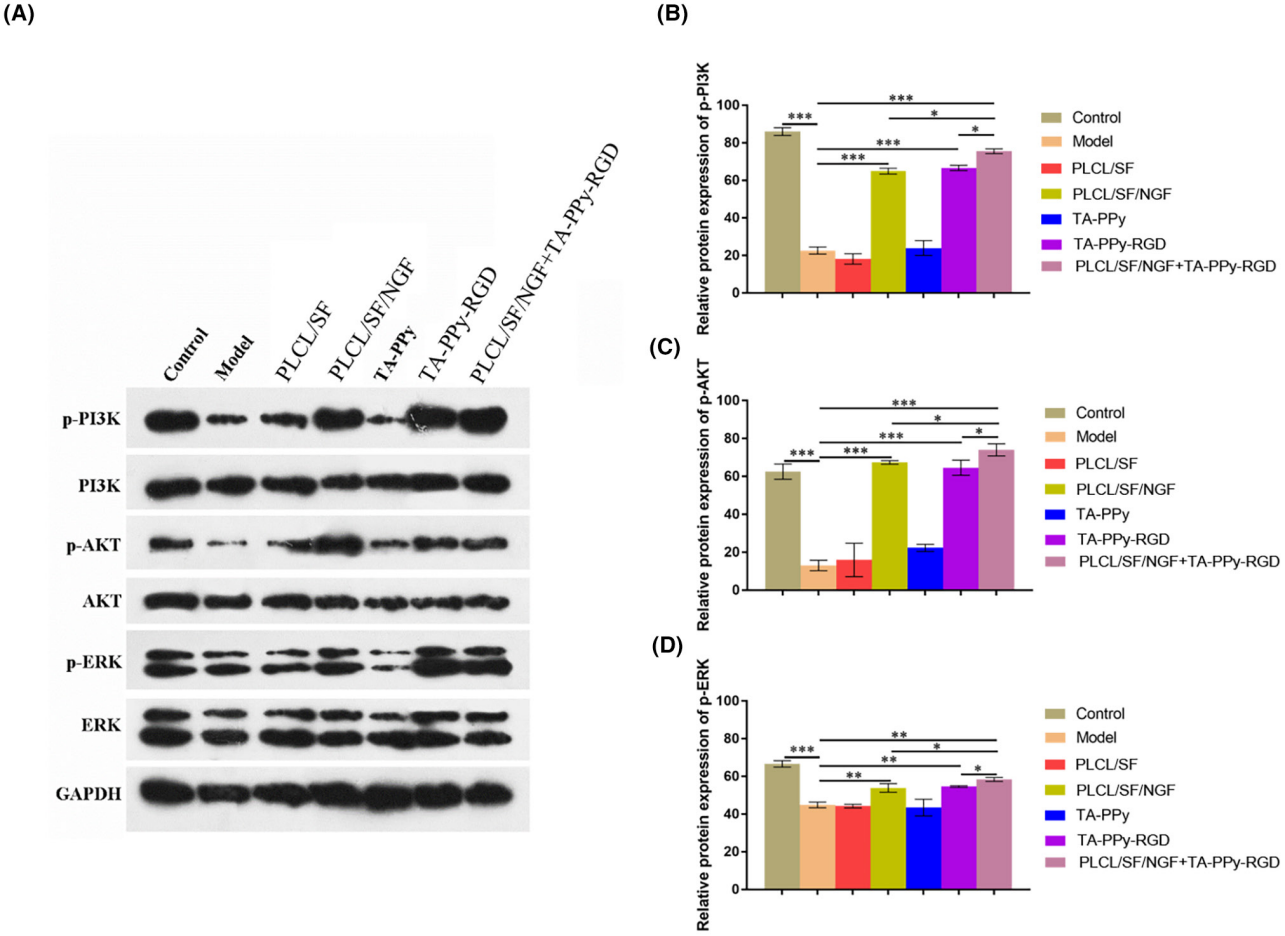
PLCL/SF/NGF and TA‐PPy‐RGD regulating the effect of H_2_O_2_ on the PC12 cell by activating the PI3K/AKT signalling pathway. To further investigate whether the protective effects of PLCL/SF/NGF and TA‐PPy‐RGD on PC12 cell damage are related to the PI3K/Akt signalling pathway. After co‐culture with PLCL/SF/NGF and/or TA‐PPy‐RGD for 48 h, (A–D) Western blot assay was used to detect PI3K and AKT signalling pathway related molecules (PI3K, AKT, ERK) and their phosphorylation level (p‐PI3K, p‐AKT, p‐ERK) on the H_2_O_2_ treated PC12 cells. NGF, nerve growth factor; PLCL, polylactic acid co caprolactone; PPy, polypyrrole; TA, tannic acid. *p < 0.05, **p < 0.01, and ***p < 0.001.

The PI3K/AKT signalling pathway plays an important role in cell survival, growth, division, and proliferation, as well as in protecting damaged neurons. To further investigate whether the protective effects of PLCL/SF/NGF and TA‐PPy‐RGD on H_2_O_2_ treated PC12 cell damage are related to the PI3K/Akt signalling pathway. After co‐culture with PLCL/SF/NGF and/or TA‐PPy‐RGD for 48 h, a western blot assay was used to detect PI3K and AKT signalling pathway related molecules and their phosphorylation level. According to results, compared to H_2_O_2_ treated PC12 cell group, the results indicate that PLCL/SF/NGF and TA‐PPy‐RGD can significantly increase the levels of p‐PI3K, p‐AKT, and p‐ERK. Moreover, PLCL/SF/NGF and TA‐PPy‐RGD have a synergistic effect. Compared with PLCL/SF/NGF and TA‐PPy‐RGD groups. The levels of p‐PI3K, p‐AKT, and p‐ERK in PLCL/SF/NGF + TA‐PPy‐RGD group were significantly increased (Figure 5A–D).

### 3.6 PLCL/SF/NGF@TA‐PPy‐RGD nerve conduit can repair and improve sciatic nerve defects in rats

**FIGURE 6:**
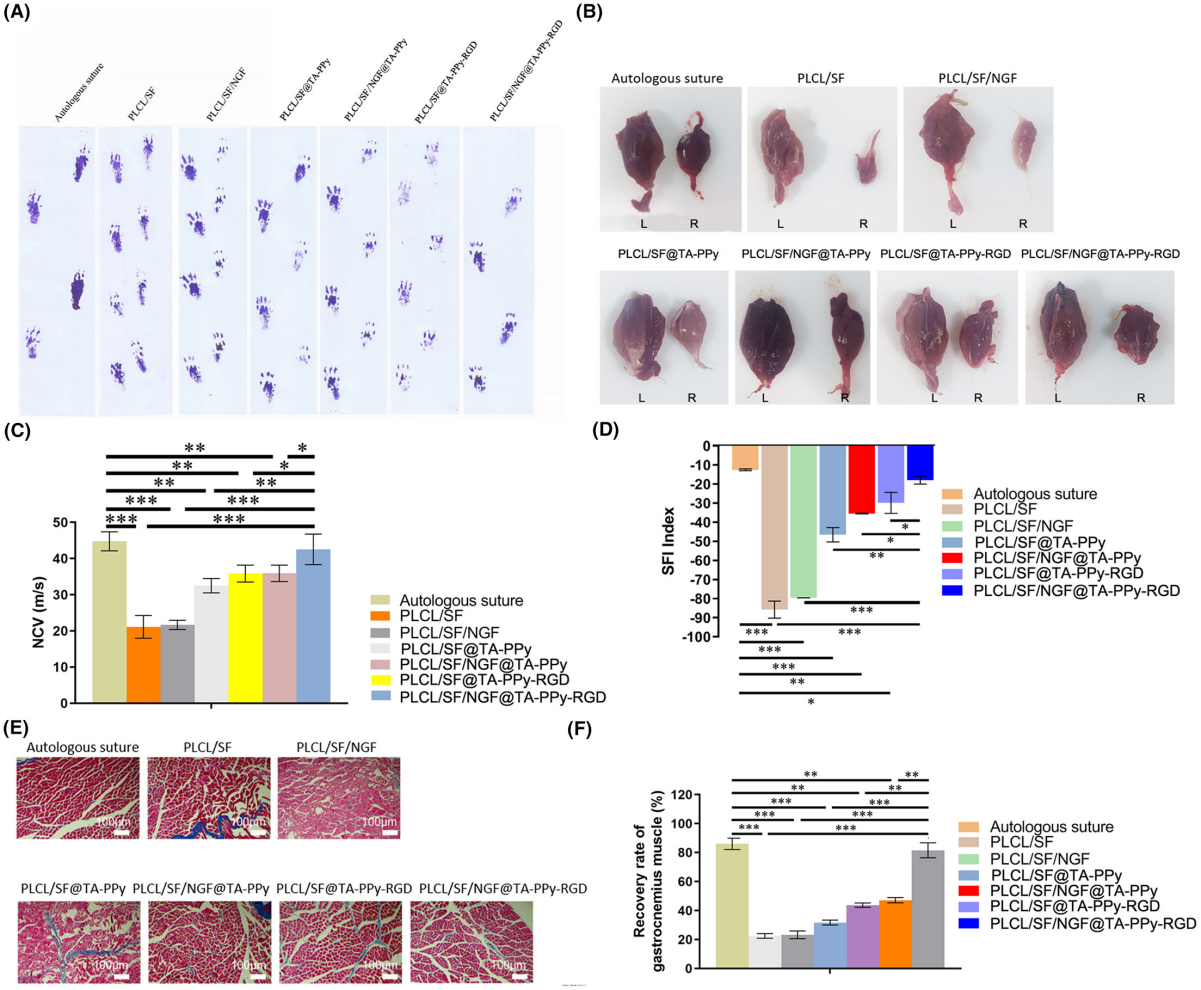
PLCL/SF/NGF@TA‐PPy‐RGD nerve conduit can repair and improve sciatic nerve defects in rats. (A) The effect of the different nerve conduits on the walking trajectory (A), gastrocnemius muscle, L represents the gastrocnemius muscle of the left leg of the rat, which is the control group, R represents the gastrocnemius muscle of the right leg of the rat, which is the model group (B, C), SFI index (D) of sciatic nerve defects rats, (E) the MASSON staining images of gastrocnemius muscle tissue slices in each group. (F) The nerve conduction rate was tested in each group. NGF, nerve growth factor; PLCL, polylactic acid co caprolactone; PPy, polypyrrole; TA, tannic acid. *p < 0.05, **p < 0.01, and ***p < 0.001.

In order to investigate the repair effect of sciatic nerve defect, a 10 mm sciatic nerve defect rat model was established on the right leg of SD rats, PLCL/SF/NGF@TA‐PPy‐RGD neural conduit was used to connect the defective nerves. 12 weeks after surgery, the defective nerve was successfully connected in the PLCL/SF/NGF@TA‐PPy‐RGD neural conduit. As autologous transplantation is the gold standard for treating nerve defects in clinical practice, we used the autologous transplantation group as a positive control in in vivo experiments. We observed the recovery of sciatic nerve injury by analysing the walking trajectory (Figure 6A), the gastrocnemius muscle recovery (Figure 6B,C), SFI index (Figure 6D) of SD rat model of sciatic nerve injury. According to the results, compare to the PLCL/SF group, the PLCL/SF@TA‐PPy, PLCL/SF@TA‐PPy‐RGD and PLCL/SF/NGF@TA‐PPy groups can significantly improved the SFI index, increased the proportion of wet weight of the gastrocnemius muscle, and significantly improved the repair. Due to the synergistic effect of NGF and RGD, the PLCL/SF/NGF@TA‐PPy‐RGD group showed a significantly better repair effect compared with the PLCL/SF@TA‐PPy‐RGD and PLCL/SF/NGF@TA‐PPy groups, Figure 6E shows the image of gastrocnemius muscle tissue slices after MASSON staining. The results of nerve conduction rate test showed that PLCL/SF and PLCL/SF/NGF did no significantly improve nerve conduction rate. However, the PLCL/SF@TA‐PPy, PLCL/SF/NGF@TA‐PPy, PLCL/SF/NGF@TA‐PPy‐RGD, and PLCL/SF/NGF@TA‐PPy‐RGD groups significantly improved nerve conduction rate, with PLCL/SF/NGF@TA‐PPy‐RGD group having a better effect than the Autologous suture group (Figure 6F).

### 3.7 PLCL/SF/NGF@TA‐PPy‐RGD neural conduit can significantly increase angiogenesis of defective nerves

**FIGURE 7:**
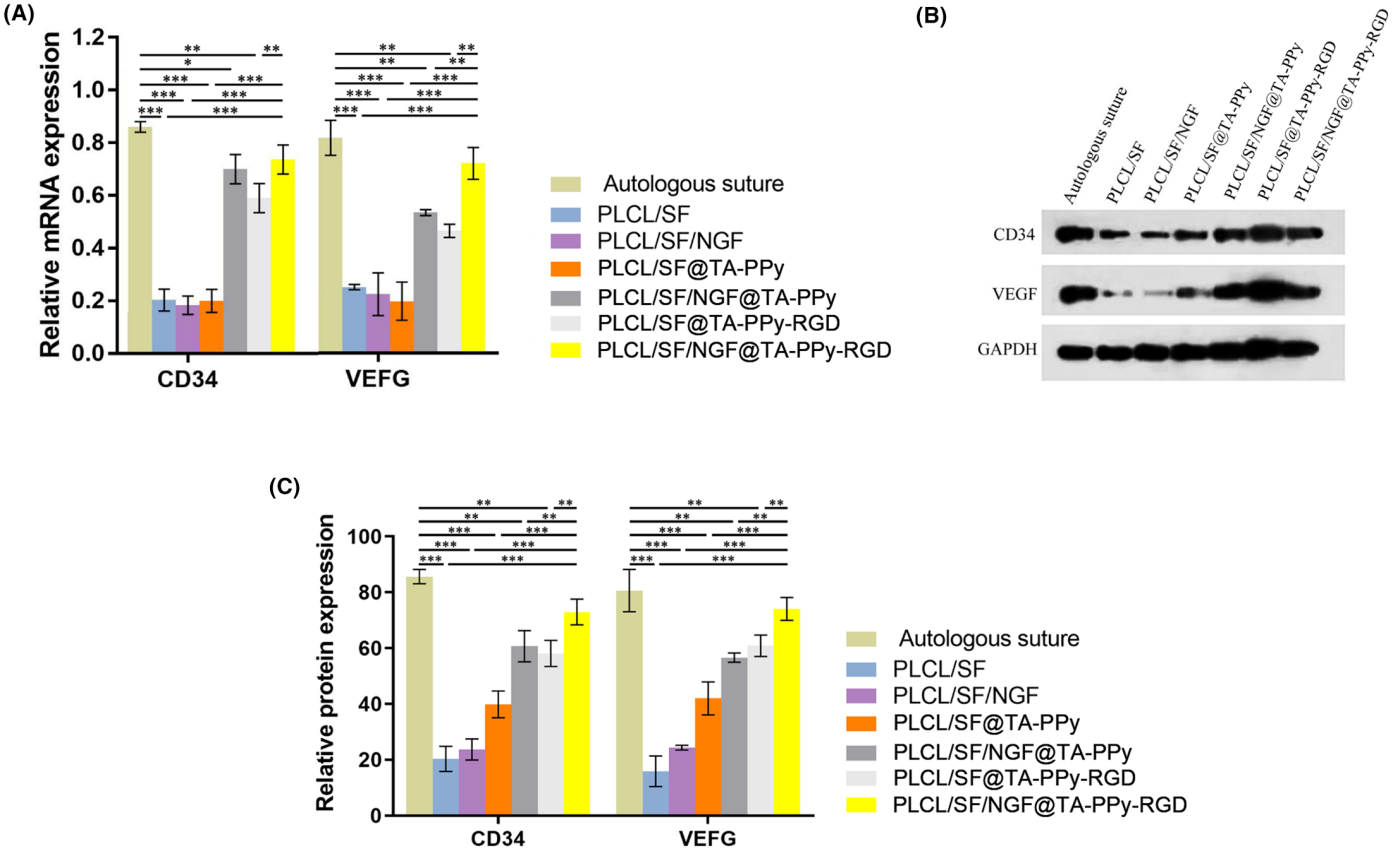
PLCL/SF/NGF@TA‐PPy‐RGD neural conduit can significantly increase angiogenesis of defective nerves. Microangiogenesis is crucial for nerve regeneration, providing nutrients and guiding the migration of nerve cells and axonal regeneration. CD34 is an endothelial marker of vascular distribution, and vascular endothelial growth factor (VEGF) is an important angiogenic factor in regenerative tissue engineering. qPCR assay (A) and Western blot assay (B, C) were used to detect the levels of CD34 and VEGF in the neural tissue. NGF, nerve growth factor; PLCL, polylactic acid co caprolactone; PPy, polypyrrole; TA, tannic acid. *p < 0.05, **p < 0.01, and ***p < 0.001.

Microangiogenesis is crucial for nerve regeneration, providing nutrients and guiding the migration of nerve cells and axonal regeneration. CD34 is an endothelial marker of vascular distribution, and vascular endothelial growth factor (VEGF) is an important angiogenic factor in regenerative tissue engineering. Here, qPCR and Western blot assay were used to detect the levels of CD34 and VEGF in the neural tissue. PLCL/SF@TA‐PPy, PLCL/SF@TA‐PPy‐RGD, and PLCL/SF/NGF@TA‐PPy can significantly increase the levels of CD34 and VEGF in sciatic nerve of defect rats. Due to the effects of NGF and RGD, the levels of CD34 and VEGF were significantly higher in PLCL/SF@TA‐PPy‐RGD and PLCL/SF/NGF@TA‐PPy group compare to the PLCL/SF@TA‐PPy group. As the synergistic effect of NGF and RGD, compared with PLCL/SF@TA‐PPy‐RGD and PLCL/SF/NGF@TA‐PPy group, PLCL/SF/NGF@TA‐PPy‐RGD group can significantly increase the levels of CD34 and VEGF (Figure 7A–C).

### 3.8 PLCL/SF/NGF@TA‐PPy‐RGD neural conduit can significantly increase the NF‐200 and MBP

**FIGURE 8:**
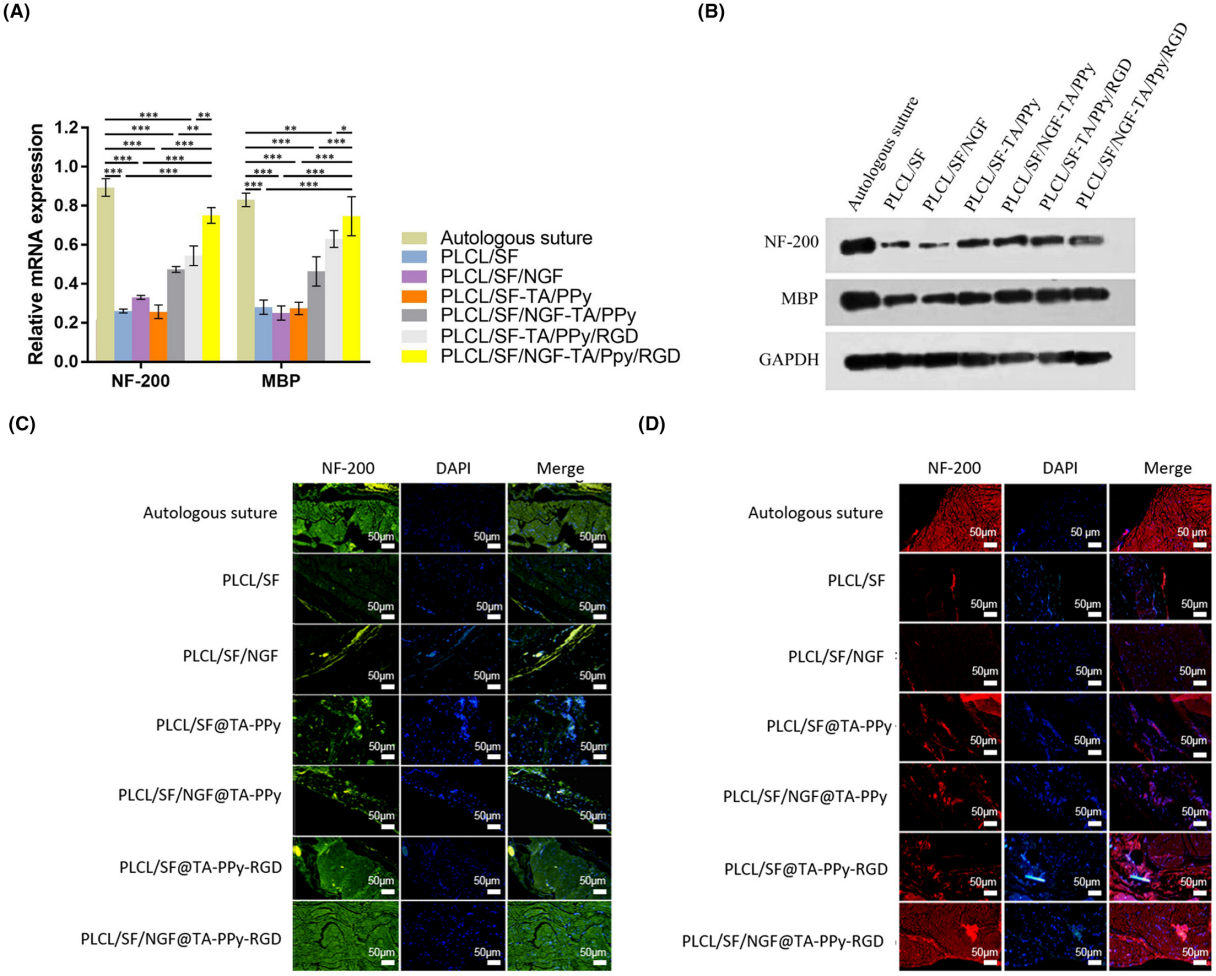
PLCL/SF/NGF@TA‐PPy‐RGD neural conduit can significantly increase the NF‐200 and MBP. The levels of neurofibrillary marker protein NF‐200 and myelin marker protein MBP are important factors of nerve injury repair. qPCR assay (A), western blot assay (B) and immunofluorescence assay (C, D) was used to detect the NF‐200 and MBP level in the neural tissue of rat models. NGF, nerve growth factor; PLCL, polylactic acid co caprolactone; PPy, polypyrrole; TA, tannic acid. *p < 0.05, **p < 0.01, and ***p < 0.001.

The levels of neurofibrillary marker protein NF‐200 and myelin marker protein MBP are important factors of nerve injury repair. Here, qPCR (Figure 8A), western blot (Figure 8B) and immunofluorescence (Figure 8C,D) assays were used to detect the NF‐200 and MBP level in the neural tissue of rat models. PLCL/SF@TA‐PPy, PLCL/SF@TA‐PPy‐RGD and PLCL/SF/NGF@TA‐PPy group can significantly increase the levels of NF‐200 and MBP in sciatic nerve defect rats, with the effects of NGF and RGD, both PLCL/SF@TA‐PPy‐RGD and PLCL/SF/NGF@TA‐PPy can significantly increase the NF‐200 and MBP compared to PLCL/SF@TA‐PPy. As PLCL/SF/NGF@TA‐PPy‐RGD has synergistic effects of NGF and RGD, it can significantly increase the levels of NF‐200 and MBP in sciatic nerve defect rats compared with PLCL/SF@TA‐PPy‐RGD and PLCL/SF/NGF@TA‐PPy group.

## 4 DISCUSSION

The main function of the peripheral nervous system is to connect peripheral receptors and effectors with the central nervous system, assisting the central nervous system in receiving information and executing action commands.^2^, ^24^ PNI is a common clinical injury. The incidence rate of PNI is high, accounting for 2.8% of trauma patients. At least 2 million people worldwide suffer from PNI every year.^25^, ^26^ In recent years, the number of cases of nerve defects has been rising, and nerve defects have a high disability rate, affecting the quality of life of patients, and also bringing heavy medical and economic burdens to society. PNI with a smaller degree of defect can be repaired by nerve end‐to‐end suturing, while nerve defects with a longer distance require autologous secondary nerves (usually sensory nerves) such as the sural nerve as donors to be transplanted to the nerve defect site, in order to reconstruct nerve function to a certain extent.^27^, ^28^ However, autologous transplantation surgery requires the destruction of the autologous nerve, and the source of the donor nerve is limited. The length and size of the nerve need to be matched, and it may cause loss of function in the donor area, and there may be risks such as post‐transplant immune response, which limits its clinical application.^15^, ^29^ With the development of tissue engineering technology, tissue engineering neural conduits have become one of the important means to solve the problems of peripheral nerve defects and repair.^30^, ^31^, ^32^ High voltage electrospinning technology has advantages such as easy moulding, simple operation, and the prepared materials have a nanofiber structure. Previous studies have successfully constructed neural conduits using this technology, which can guide the regeneration, repair, and functional reconstruction of damaged nerves.^33^ The structure of the nerve conduit mainly includes the conduit shell and the substance inside the conduit, of which the conduit shell mainly plays the role of protecting nerves and bridging, and the gel inside the conduit mainly plays the role of the transmission of nerve electrical signals and the role of the nerve cell proliferation site.^33^

Here, we use the electrospinning technology to prepare the PLCL/SF containing NGF into a nanofiber film as the nerve conduit shell material, which not only has good biocompatibility, but it can also slowly release NGF, promoting the proliferation of nerve cells. We used TA‐PPy‐RGD conductive gel as the inner core of the nerve conduit. TA‐PPy‐RGD contributes to nerve regeneration by providing a bioactive and conductive environment that supports cell adhesion, proliferation, and differentiation. The RGD peptides enhance cell attachment, while the conductive properties of PPy facilitate electrical signalling that is crucial for nerve function. We have also added corresponding textual explanations in the revised article. The results showed that TA‐PPy‐RGD had good biocompatibility, and could provide ideal anchor points for nerve cells to promote the proliferation of nerve cells. TA‐PPy‐RGD also has good conductivity, providing a good conductive medium for the transmission of neural electrical signals. Subsequently, we tested the effects of the constructed neural substances on the function of H_2_O_2_ damaged nerve cells at both in vitro and in vivo levels. The results showed that PLCL/SF/NGF and TA‐PPy‐RGD not only significantly increased the activity, proliferation, and migration ability of PC12 cells, but also significantly reduced neuronal apoptosis in H_2_O_2_ damaged cells. PLCL/SF/NGF and TA‐PPy‐RGD have a synergistic effect. The PLCL/SF/NGF + TA‐PPy‐RGD group can significantly increase the activity, proliferation, and migration ability of PC12 cells compared to the PLCL/SF/NGF and TA‐PPy‐RGD groups, and significantly reduce neuronal apoptosis. The PI3K/AKT signalling pathway is crucial in nerve regeneration, influencing cell survival, growth, and proliferation. The study demonstrates that PLCL/SF/NGF and TA‐PPy‐RGD enhance this pathway, increasing levels of phosphorylated PI3K, AKT, and ERK in H_2_O_2_‐treated PC12 cells. Our research shown that adding and releasing NGF in the shell and increasing RGD anchor points in the conductive gel can significantly activate the PI3K/AKT signalling pathway in injured nerve cells, while PLCL/SF/NGF + TA‐PPy‐RGD also has a synergistic effect. We tested the repairing effect of the H_2_O_2_ constructed neural material on the neural defect model in rats, and the results showed that compared with the model group, PLCL/SF@TA‐PPy, PLCL/SF@TA‐PPy‐RGD and PLCL/SF/NGF@TA‐PPy groups can significantly improve the SFI index, the proportion of wet weight increase in the gastrocnemius muscle, and the repair of nerve tissue in a rat model of sciatic nerve defect, Due to the synergistic effect of NGF and RGD, PLCL/SF@TA‐PPy‐RGD and PLCL/SF/NGF@TA‐PPy has better repair effect. Microangiogenesis is crucial for nerve regeneration, providing nutrients for nerve regeneration, guiding the migration of nerve cells, and axonal regeneration. CD34 is an endothelial marker of vascular distribution, and VEGF is an important angiogenic factor in regenerative tissue engineering. The immunostaining of these proteins on regenerated nerve sections can further confirm the relationship between angiogenesis and Schwann cell proliferation. Our research findings indicate that PLCL/SF@TA‐PPy, PLCL/SF@TA‐PPy‐RGD and PLCL/SF/NGF@TA‐PPy significantly increased the levels of CD34 and VEGF in sciatic nerve defect rats, Due to the effects of NGF and RGD, CD34 and VEGF levels in PLCL/SF@TA‐PPy‐RGD and PLCL/SF/NGF@TA‐PPy group was significantly higher than PLCL/SF@TA‐PPy group, PLCL/SF/NGF@TA‐PPy‐RGD group has a synergistic effect of NGF and RGD, the CD34 and VEGF was significantly higher than that in PLCL/SF@TA‐PPy‐RGD and PLCL/SF/NGF@TA‐PPy group. The levels of neurofibrillary marker protein NF‐200 and myelin marker protein MBP are important indicators of nerve injury repair. Here, qPCR and western blot assay was used to detect the levels of NF‐200 and MBP in the neural tissue of rat models. Compared with the model group, PLCL/SF@TA‐PPy, PLCL/SF@TA‐PPy‐RGD and PLCL/SF/NGF@TA‐PPy group can significantly increase the levels of NF‐200 and MBP in sciatic nerve defect rats, with the effects of NGF and RGD, PLCL/SF@TA‐PPy‐RGD and PLCL/SF/NGF@TA‐PPy can significantly increase the NF‐200 and MBP compare to PLCL/SF@TA‐PPy. As PLCL/SF/NGF@TA‐PPy‐RGD has synergistic effect of NGF and RGD, it can significantly increase the levels of NF‐200 and MBP in sciatic nerve defect rats compare with PLCL/SF@TA‐PPy‐RGD and PLCL/SF/NGF@TA‐PPy group. The PLCL/SF/NGF@TA‐PPy‐RGD nerve conduit prepared here presents a significant advancement in nerve regeneration technology by combining biocompatibility, bioactivity, and electrical conductivity.^34^, ^35^ The unique combination of materials in the current study, including the CP TA‐PPy‐RGD within a PLCL/SF scaffold, represents a significant innovation. This combination not only leverages the biocompatibility and structural benefits of PLCL/SF but also introduces the electrical conductivity of TA‐PPy‐RGD, which can further enhance nerve cell proliferation and migration by mimicking the electrical properties of neural tissue. Moreover, the modification of TA‐PPy with RGD peptides introduces a bioactive component that can significantly enhance cell adhesion and proliferation, addressing one of the common challenges in neural conduit design: the promotion of cell‐material interactions to support nerve regeneration.^36^ This study's approach to integrating NGF directly into the conduit design also sets it apart, providing a sustained release of NGF to support nerve repair over time, a feature that has been highlighted as crucial for the success of neural conduits.^19^, ^37^ The PLCL/SF/NGF@TA‐PPy‐RGD neural conduit represents a significant advancement in neural conduit design, incorporating conductivity, bioactivity, and controlled release of growth factors. These features collectively aim to address the limitations observed in previous designs, potentially offering a more effective solution for peripheral nerve regeneration. The PLCL/SF/NGF@TA‐PPy‐RGD nerve conduit utilizes a PLCL/SF scaffold known for its excellent biocompatibility, which minimizes immune reactions and promotes cell adhesion and proliferation. The incorporation of NGF within the scaffold allows for sustained release of growth factors, crucial for enhancing nerve regeneration over time. Additionally, the inclusion of TA‐PPy‐RGD introduces electrical conductivity, mimicking the natural electrical properties of neural tissue, which is essential for enhancing nerve cell proliferation and migration. These features collectively promote cell proliferation and migration significantly more effectively than other conduits. Comparatively, traditional nerve conduits such as those made from chitosan, collagen, and synthetic polymers (PGA and PLGA) have their own benefits but fall short in some critical areas. Chitosan conduits, while excellent in promoting cell adhesion and proliferation, lack the electrical conductivity offered by the PLCL/SF/NGF@TA‐PPy‐RGD conduit. Collagen conduits are biocompatible and mechanically supportive but do not provide sustained growth factor release or electrical properties. Synthetic polymer conduits, though strong and supportive, also lack bioactive components and electrical conductivity. Composite materials like PHBV‐PLGA offer adjustable mechanical properties and degradation rates, providing a versatile platform for nerve regeneration. However, they do not incorporate electrical conductivity or sustained growth factor release, limiting their overall efficacy in nerve repair. The PLCL/SF/NGF@TA‐PPy‐RGD conduit stands out by addressing these limitations. It promotes enhanced cell proliferation, migration, and survival through the synergistic effects of NGF and RGD peptides, which improve cell adhesion and proliferation. Additionally, it activates the PI3K/AKT signalling pathway, crucial for cell survival and growth. Despite these advancements, there is a need for further optimization in the dosage and delivery mechanisms of NGF and RGD, and more studies are required to explore the long‐term effects and clinical applications of this conduit. In conclusion, the PLCL/SF/NGF@TA‐PPy‐RGD conduit demonstrates superior capabilities in nerve regeneration compared to traditional and composite nerve conduits. Its innovative combination of biocompatibility, bioactivity, and electrical conductivity makes it a promising solution for comprehensive nerve repair, highlighting the need for continued research and development to fully realize its potential in clinical settings. This study is limited to cell experiments and animal models, and there are still various challenges in applying the research results to clinical practice. Strict regulatory barriers, detailed preclinical and clinical data, and the transition from laboratory scale production to large‐scale production need to be addressed to ensure high quality and consistency. Although the results of animal models are encouraging, adverse reactions such as immune reactions and long‐term biocompatibility issues may occur in the human body. Therefore, long‐term follow‐up studies are crucial. Future research should explore PLCL/SF in depth/NGF@TA‐PPy‐RGD Conduct multicentre, large‐scale, and long‐term randomized controlled trials to evaluate the molecular and cellular mechanisms of neural conduits, evaluate their efficacy and safety, develop personalized treatment plans, and compare and analyse them with existing neural conduits. By collaborating with regulatory agencies in the early stages, simplify the approval process and ensure the effectiveness of data collection and presentation.

## 5 CONCLUSION

PNI has a higher disability rate. At present, the gold standard for nerve defects is autologous nerve transplantation, but surgery is required to remove the autologous nerve. The source of the donor nerve is limited, and the length and size of the nerve need to be matched. This inevitably leads to functional loss of the donor area, resulting in nerve tumours, scars, and even donor site infections at the residual end of the donor nerve. To address the limitations of autologous nerve transplantation, we first utilized electrospinning technology and conductive gel to construct a PLCL/SF catheter loaded with NGF as the shell and incorporated a TA‐PPy conductive gel modified with RGD polypeptide inside the catheter. Ultimately, we constructed PLCL/SF/NGF@TA‐PPy‐RGD neural conduit that slowly release NGF to promote nerve cell growth, and on the other hand, RGD can provide growth anchor points for nerve cells, which can also promote the repair of missing nerves. Both in vivo and in vitro experimental results indicate that PLCL/SF/NGF@TA‐PPy‐RGD significantly promotes the proliferation and migration of nerve cells damaged by H_2_O_2_, and significantly inhibit the apoptosis of those cells. PLCL/SF/NGF@TA‐PPy‐RGD neural conduits demonstrated a significant repairing effect on nerve defects in rats. In summary, this paper provides a useful theoretical basis for the treating nerve defects and proposes a preclinical research plan based on neural conduit therapy for nerve defects. However, its generalizability is limited by a single animal model and a limited sample size. Comprehensive follow‐up experiments are needed to prove the long‐term biocompatibility, stability, and neural recovery. In addition, further exploration and research are needed on the molecular mechanisms of neural conduit repair for damaged nerves. Advancing this neural conduit technology can provide a less invasive alternative to traditional nerve transplantation, and the success of this study can reduce the economic and emotional losses of patients by improving the efficiency of nerve repair, potentially changing the treatment pattern of nerve injury.

## AUTHOR CONTRIBUTIONS

**Kunyu Liu:** Conceptualization (equal); data curation (equal); formal analysis (equal); investigation (equal); methodology (equal); project administration (equal); resources (equal); software (equal); supervision (equal); validation (equal); visualization (equal); writing – original draft (equal); writing – review and editing (equal). **Weilong Tang:** Conceptualization (equal); methodology (equal); validation (equal). **Shixin Jin:** Project administration (equal); writing – original draft (equal). **Xin Hao:** Conceptualization (equal); data curation (equal). **Yuhang Hu:** Software (equal); writing – original draft (equal). **Tianyi Zhou:** Software (equal); writing – review and editing (equal). **Chenliang Zhou:** Formal analysis (equal); software (equal). **Guanghua Chen:** Validation (equal); visualization (equal). **Yifeng Cui:** Resources (equal); software (equal); validation (equal). **Qianqi Liu:** Investigation (equal); resources (equal); writing – original draft (equal). **Zhenyu Zhang:** Conceptualization (equal); data curation (equal); formal analysis (equal); funding acquisition (equal); investigation (equal); methodology (equal); project administration (equal); visualization (equal).

## FUNDING INFORMATION

This work was supported by grant 2020HX018 from the Ren Xin Medical Relief Fund.

## CONFLICT OF INTEREST STATEMENT

The authors declare that the research was conducted in the absence of any commercial or financial relationships that could be construed as a potential conflict of interest.

## Data Availability

The authors declare that the research was conducted in the absence of any commercial or financial relationships that could be construed as a potential conflict of interest.
